# Microbiome and Resistome in Poultry Litter-Fertilized and Unfertilized Agricultural Soils

**DOI:** 10.3390/antibiotics14040355

**Published:** 2025-03-31

**Authors:** Eliene dos Santos Lopes, Larissa Coutinho Araujo de Souza, Karen Caroline Ferreira Santaren, Cláudio Ernesto Taveira Parente, Lucy Seldin

**Affiliations:** 1 Laboratory of Microbial Genetics, Institute of Microbiology Paulo de Góes, Federal University of Rio de Janeiro (UFRJ), Rio de Janeiro 21941-590, RJ, Brazil; elienelopestst@micro.ufrj.br (E.d.S.L.); laricoutinhobio@ufrrj.br (L.C.A.d.S.); karensantaren@micro.ufrj.br (K.C.F.S.); 2 Olaf Malm Environmental Studies Laboratory, Carlos Chagas Filho Institute of Biophysics, Federal University of Rio de Janeiro (UFRJ), Rio de Janeiro 21941-590, RJ, Brazil; cparente@biof.ufrj.br

**Keywords:** agricultural soil, antimicrobial-resistant bacteria, chicken manure, poultry farming, environmental health, resistance genes

## Abstract

**Background**: Poultry litter is the main waste of poultry farming and is widely used as an agricultural fertilizer. However, owing to the use of antimicrobials in animal production, it can accumulate antimicrobial residues, antimicrobial-resistant bacteria (ARB), and antimicrobial resistance genes (ARGs). This study aimed to evaluate the impact of poultry litter use on the microbiome and resistome of agricultural soils. **Methods**: Soil samples from fertilized and unfertilized plots were collected from two horticultural farms that intensively use poultry litter. Microbiome composition was assessed using 16S rRNA sequencing. A culture-dependent method was used to isolate resistant strains on CHROMagar plates supplemented with sulfamethoxazole or ciprofloxacin. ARGs and integrase-encoding genes were identified by PCR. **Results**: Microbiome analysis revealed significant differences in structure and composition between poultry litter-fertilized and unfertilized soils. Fertilized soils exhibited greater alpha diversity and richness. Bacillota, commonly found in the avian gastrointestinal tract, were more abundant in fertilized soils. A total of 62 resistant strains were isolated, and 23 clinically relevant strains harbored ARGs, including fluoroquinolone (*qnrA* and *qnrB*) and β-lactam (*bla*_GES_, *bla*_TEM_, and *bla*_SHV_) resistance genes. Class 1 and 2 integron-associated genes (*intI1* and *intI2*) were also detected. Notably, the rare *bla*_GES_ gene was detected in *Bacillus* sp. from unfertilized soil. Similarly, *qnrA* co-occurred with *bla*_SHV_ in a *Bosea* sp. strain from unfertilized soil. **Conclusions**: These findings highlight the potential for ARB dissemination in agricultural environments, where ARB and ARGs, once introduced into soils, may spread by weathering and other environmental factors, complicating negative control selection in in situ studies.

## 1. Introduction

The use of manure as an organic fertilizer is a widely adopted agricultural practice aimed at improving soil organic matter and supplying essential nutrients to crops [1,2,3]. Among organic fertilizers, poultry litter is particularly valued for its high concentrations of macronutrients, such as nitrogen (N), phosphorus (P), and potassium (K) [4,5]. This material consists of plant-based substrates used as bedding in poultry houses, where it absorbs and accumulates excreta, feathers, water, feed remnants, and veterinary residues throughout the production cycle [1,4]. As a result, poultry litter can enhance the physical, chemical, and biological properties of agricultural soils, making it a sustainable alternative to inorganic fertilizers [4,5].

However, despite these agronomic benefits, the use of poultry litter as fertilizer raises concerns regarding microbiological safety, particularly due to the widespread use of antimicrobials in livestock production. These drugs are commonly administered to food-producing animals for therapeutic, prophylactic, and metaphylactic purposes, as well as for growth promotion, although the latter practice has been regulated or banned in several countries [6,7,8]. The continuous exposure of poultry and other livestock to these antimicrobials exerts selective pressure on their intestinal microbiota, promoting the persistence of antimicrobial-resistant bacteria (ARB) and antimicrobial resistance genes (ARGs) [8]. Furthermore, because up to 90% of the administered drug dosage can be excreted either unchanged or as bioactive metabolites, the use of untreated poultry litter as fertilizer may contribute to the dissemination of these contaminants into the environment [9,10].

Once applied to agricultural fields, poultry can serve as a vehicle for antimicrobial resistance (AMR) dissemination. Antimicrobial residues, ARB, and ARGs may persist in fertilized soils, be taken up by crops, and reach surface and groundwater through leaching and runoff [11,12]. These pathways contribute to the spread of resistant bacteria to human and animal populations, whether through direct environmental exposure, contaminated water sources, or the consumption of agricultural products grown in affected soils [12,13,14]. Given that AMR is recognized as one of the most critical global public health challenges of the 21st century, understanding its environmental dissemination is highly important [15,16].

Despite the growing recognition of AMR as a major threat, the extent to which agricultural environments contribute to its spread and potential impact on public health remains insufficiently understood [17]. The soil resistome, in particular, is considered one of the largest yet least explored reservoirs of AMR [18]. Previous studies have demonstrated the presence of ARB and ARGs in agricultural settings. Lopes et al. [19], for example, detected ARB and ARGs to sulfonamides and β-lactams in irrigation water from agricultural areas with intensive poultry litter application, highlighting the potential role of irrigation water as a vector for AMR dissemination. Subsequently, Lopes et al. [12] expanded this investigation by analyzing not only water but also other agricultural substrates. In this study, ARB and ARGs associated with resistance to sulfonamides, β-lactams, and fluoroquinolones were identified in poultry litter, soil fertilized with poultry litter, rhizospheres, pond water, and sediments. This study focused on comparing the microbiome and resistome across different agricultural substrates, providing a broader understanding of AMR dissemination in the environment.

Unlike previous studies, which often focus on controlled environments or isolated samples, the present study aims to investigate whether there are differences in microbiome structure and composition between poultry litter-fertilized and unfertilized soils in situ within real agricultural areas. In addition, it seeks to characterize the ARB and ARGs present in these soils to further elucidate the impact of poultry litter fertilization on the soil microbiota and AMR dissemination. This study hypothesizes that poultry litter fertilization significantly alters the soil microbial composition, increasing the abundance and diversity of ARB and ARGs compared to unfertilized soils. To test this hypothesis, the microbiome structure and resistome profiles of fertilized and unfertilized soils were analyzed using 16S rRNA gene sequencing and PCR-based detection of ARGs. In addition, resistant bacterial strains were isolated from both soil types to assess their antimicrobial resistance profiles.

## 2. Results

### 2.1. Antimicrobial-Resistant Bacteria Isolated from Agricultural Soils

**Figure 1:**
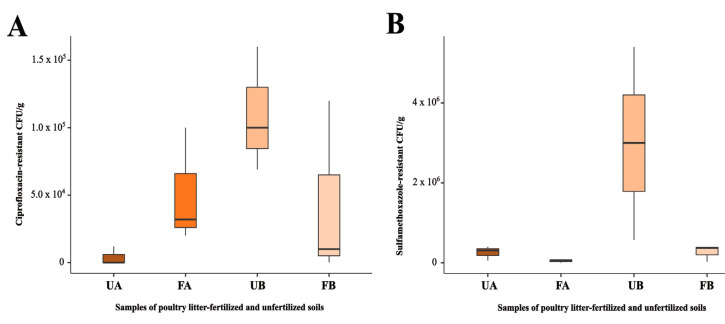
Determination of CFU/g in different soil types: poultry litter-fertilized (FA and FB) and unfertilized (UA and UB) soils from Farm A and Farm B. Samples were plated on CHROMagar supplemented with (**A**) ciprofloxacin and (**B**) sulfamethoxazole. Bars represent standard deviations. Parametric data were analyzed using one-way ANOVA.

The highest CFU/g count on ciprofloxacin-supplemented plates was observed in fertilized soil (F) from Farm A (FA—5.1 × 10^4^ CFU/g). However, in Farm B, the highest CFU/g count on ciprofloxacin-supplemented plates was found in untreated soil (U) (UB—1.1 × 10^5^ CFU/g). Nevertheless, no significant differences were observed between the samples when these parametric data were analyzed via one-way ANOVA (*p* = 0.117) (Figure 1A).

Similarly, the highest CFU/g counts on sulfamethoxazole-supplemented plates were observed in soils that had not been fertilized with poultry litter. In Farms A and B, the highest CFU/g counts on sulfamethoxazole-supplemented plates were found in untreated soil (UA—2.6 × 10^5^ CFU/g and UB—3.0 × 10^6^ CFU/g, respectively). No significant differences were observed between the samples (*p* = 0.052) (Figure 1B).

A total of 62 bacterial strains resistant to ciprofloxacin or sulfamethoxazole were isolated from all the samples. The identification results for each strain, obtained using MALDI-TOF, are presented in the Supplementary Materials (Table S1). Twenty-one strains harbored at least one resistance gene, as well as genes encoding integrases associated with integrons, as detected by PCR. The confirmation of their identification through sequencing of the 16S rRNA gene is provided in the Supplementary Materials (Table S2).

### 2.2. Detection of Resistance Genes in Bacterial Isolates and Total DNA from the Same Agricultural Soils

PCR analysis revealed the presence of ARGs in bacterial isolates, as well as genes encoding integrases associated with integrons (Table 1). Among the fluoroquinolone resistance genes analyzed, the *qnrB* gene was detected alongside the *bla*_TEM_ gene in a *Citrobacter* sp. strain isolated from unfertilized soil at Farm B (UB). The *qnrA* gene was detected in two *Citrobacter* sp. strains isolated from poultry litter-fertilized soil at Farm B (FB), one of which also carried the *bla*_TEM_ gene. This gene was also detected in strains isolated from unfertilized soils, including the *Bosea* sp. strain from Farm A, which was positive for *bla*_SHV_, and two *Stenotrophomonas* sp. strains from Farm B. Additionally, one *Bosea* sp. strain (CSUA4.2) tested positive for *bla*_TEM_ and *bla*_SHV_.

Regarding sulfonamide resistance genes, neither *sul1* nor *sul2* were detected in any of the strains, even those that tested positive for *intI1* and *intI2*. In contrast, β-lactam resistance genes were detected in strains isolated from both poultry litter-fertilized and unfertilized soils. The *bla*_TEM_ and *bla*_SHV_ genes were the most frequently detected genes among the isolates, whereas the *bla*_GES_ gene was found only in two *Bacillus* sp. strains isolated from unfertilized soil at Farm B.

Despite the presence of ARGs in bacterial strains isolated from soil samples, these genes were not detected in the total DNA extracted from the corresponding soil samples.

### 2.3. Structure of the Bacterial Community in Poultry Litter-Fertilized and Unfertilized Soils

The sequencing data from the V3–V4 region of the 16S rRNA gene were normalized to 30,160 sequences per sample. This normalization ensured adequate coverage of the operational taxonomic units (OTUs) within the bacterial communities analyzed. The effectiveness of this approach was demonstrated by the rarefaction curves approaching a plateau (Supplementary Materials—Figure S1).

**Figure 2:**
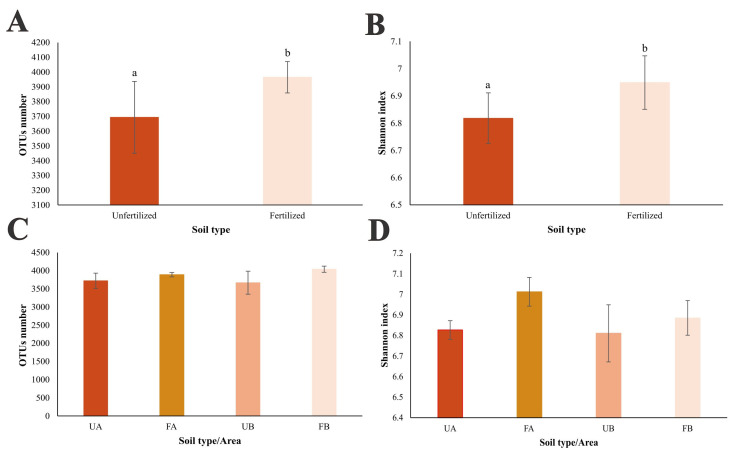
Alpha diversity was assessed through high-throughput sequencing of the 16S rRNA gene in different soil samples. (**A**) Richness, evaluated by the number of OTUs, in unfertilized soil (dark orange) and poultry litter-fertilized soil (light salmon). (**B**) Shannon diversity index for unfertilized soil (dark orange) and poultry litter-fertilized soil (light salmon). Lowercase letters indicate significant differences according to Student’s *t*-test. (**C**) Richness, evaluated by the number of OTUs, in unfertilized soil from Farm A (UA—dark orange), fertilized soil from Farm A (FA—light orange), unfertilized soil from Farm B (UB—dark salmon), and fertilized soil from Farm B (FB—light salmon). (**D**) Shannon diversity index for the same soil categories as in (**C**). Parametric data were analyzed using one-way ANOVA; nonparametric data were analyzed using the Kruskal–Wallis test. Bars represent standard deviations.

The analysis based on the comparison between fertilized and unfertilized samples with poultry litter revealed a significantly higher richness in the fertilized samples (*p* = 0.031), considering the mean number of OTUs observed (Figure 2A). A similar pattern was observed for the Shannon diversity index, indicating greater diversity in samples fertilized with poultry litter (*p* = 0.039) (Figure 2B). When the “area” factor was considered and all samples were compared using one-way ANOVA, no significant differences were observed among the samples, either in terms of richness, as indicated by the mean number of OTUs (*p* = 0.165) (Figure 2C), or in terms of diversity, as indicated by the Shannon diversity index (*p* = 0.091) (Figure 2D).

**Figure 3:**
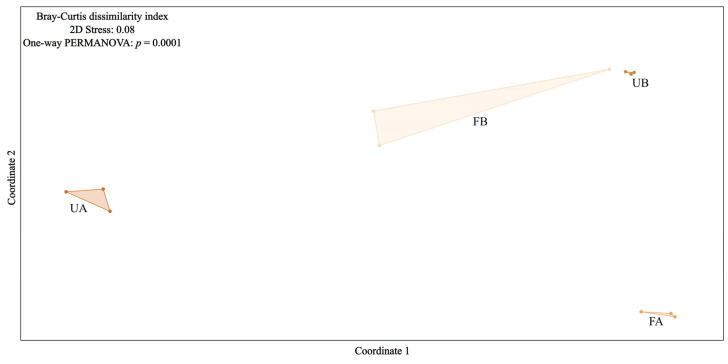
Nonmetric multidimensional scaling (NMDS) was performed via the Bray–Curtis dissimilarity index. Dark orange represents unfertilized soil (UA and UB), whereas light salmon represents poultry litter-fertilized soil (FA and FB).

NMDS ordination revealed a clear separation pattern between fertilized and unfertilized soils at Farm A. In contrast, at Farm B, the distance between the two soil types was less pronounced, suggesting greater similarity in microbiome structure between fertilized and unfertilized soils in this area. Additionally, a distinct separation between samples from Farms A and B was observed, indicating differences in bacterial community structure between the two locations. These findings were corroborated by one-way PERMANOVA, which revealed a significant difference (*p* = 0.0001) in bacterial community structure across the different samples (Figure 3).

### 2.4. Composition of the Bacterial Community in Poultry Litter-Fertilized and Unfertilized Soils

**Figure 4:**
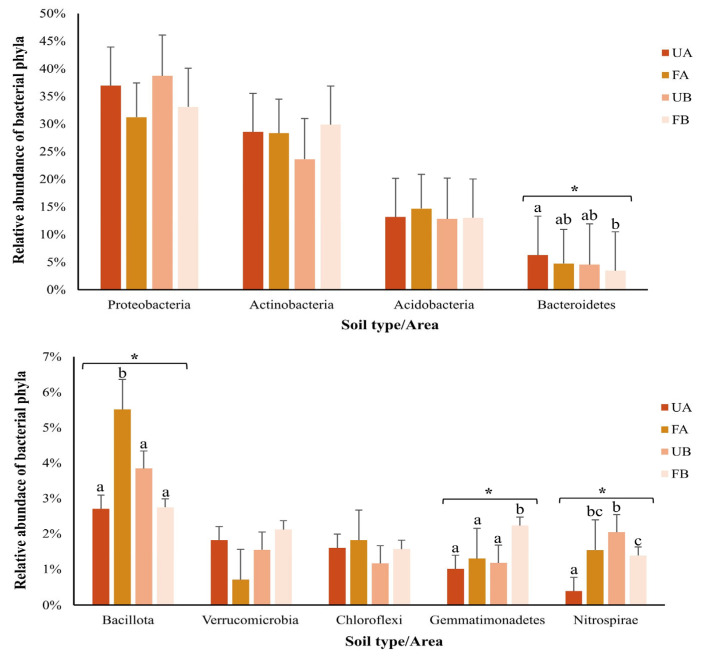
Relative abundance of bacterial phyla. The colors represent unfertilized soil from Farm A (UA—dark orange), fertilized soil from Farm A (FA—light orange), unfertilized soil from Farm B (UB—dark salmon), and fertilized soil from Farm B (FB—light salmon). The bars represent the standard deviation. Asterisks indicate significant differences among samples (parametric data were analyzed using one-way ANOVA; nonparametric data were analyzed using the Kruskal–Wallis test). Letters denote significant differences among samples, as determined by post hoc tests (Tukey’s test or Dunn’s test).

Approximately 92% of the sequences obtained from 16S rRNA gene sequencing were classified at the phylum level, encompassing 21 phyla. The remaining 8% could not be classified at this taxonomic level and were assigned only to the domain Bacteria. The most abundant phyla across all the samples, each comprising at least 1% of the classified OTUs, are represented in Figure 4. The relative abundances of the phyla Bacteroidetes, Bacillota, Gemmatimonadetes, and Nitrospirae differed significantly (*p* < 0.05) among the samples, as determined by one-way ANOVA. Tukey’s test revealed a higher abundance of Bacillota in poultry litter-fertilized soil from Farm A and a higher abundance of Gemmatimonadetes in poultry litter-fertilized soil from Farm B.

**Figure 5:**
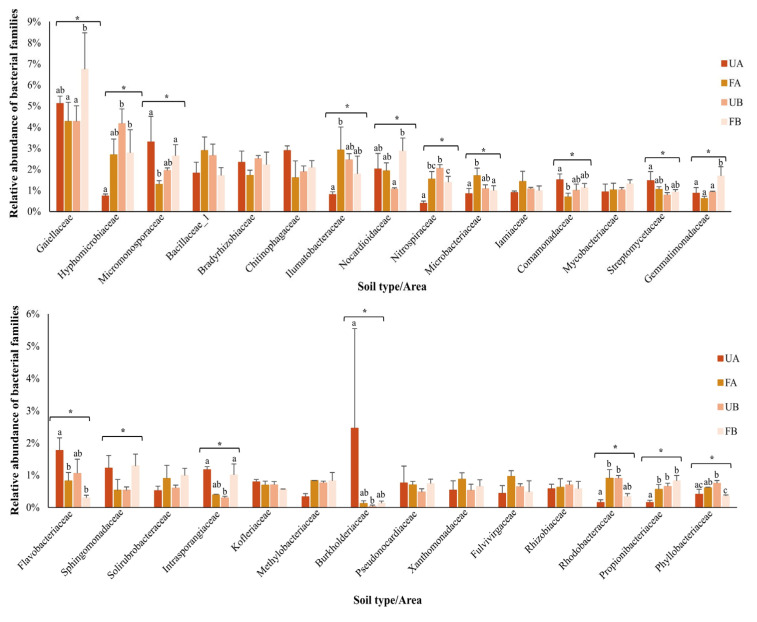
Relative abundance of bacterial families. The colors represent unfertilized soil from Farm A (UA—dark orange), fertilized soil from Farm A (FA—light orange), unfertilized soil from Farm B (UB—dark salmon), and fertilized soil from Farm B (FB—light salmon). The bars represent the standard deviation. Asterisks indicate significant differences among samples (parametric data were analyzed using one-way ANOVA; nonparametric data were analyzed using the Kruskal–Wallis test). Letters denote significant differences among samples, as determined by post hoc tests (Tukey’s test or Dunn’s test).

Forty-nine percent of the obtained sequences were assigned at the family level, totaling 226 identified families. The most abundant families across all the samples, each representing at least 1% of the classified OTUs, are shown in Figure 5. Significant differences (*p* < 0.05) were observed in the relative abundances of the families *Gaiellaceae*, *Hyphomicrobiaceae*, *Micromonosporaceae*, *Ilumatobacteraceae*, *Nocardioidaceae*, *Nitrospiraceae*, *Microbacteriaceae*, *Comamonadaceae*, *Streptomycetaceae*, *Gemmatimonadaceae*, *Flavobacteriaceae*, *Sphingomonadaceae*, *Intrasporangiaceae*, *Burkholderiaceae*, *Rhodobacteraceae*, *Propionibacteriaceae*, and *Phyllobacteriaceae*. However, no significant differences were found in the composition of Sphingomonadaceae among the samples when analyzed using Tukey’s test. Post hoc comparisons (Tukey’s test or Dunn’s test) revealed significant differences in the relative abundances of specific families between poultry litter-fertilized and unfertilized soils. In Farm A, *Micromonosporaceae*, Comamonadaceae, and Flavobacteriaceae were significantly more abundant in unfertilized soil, whereas Ilumatobacteraceae, *Nitrospiraceae*, *Microbacteriaceae*, *Rhodobacteraceae*, and *Propionibacteriaceae* were significantly more abundant in fertilized soil. In Farm B, *Nitrospiraceae* and *Phyllobacteriaceae* were significantly more abundant in unfertilized soil, whereas *Gaiellaceae*, *Nocardioidaceae*, *Gemmatimonadaceae*, and *Intrasporangiaceae* were significantly more abundant in fertilized soil.

**Figure 6:**
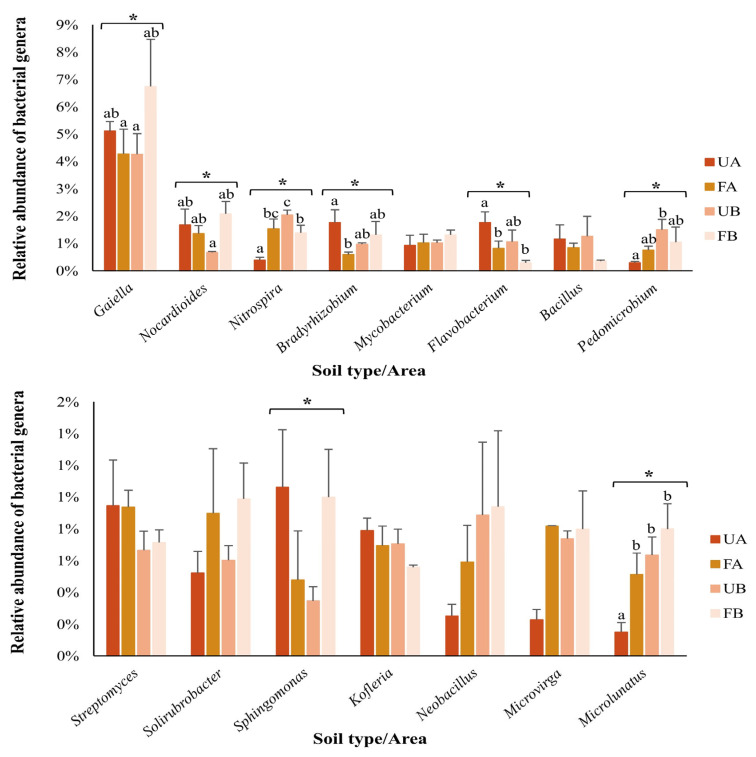
Relative abundance of bacterial genera. The colors represent unfertilized soil from Farm A (UA—dark orange), fertilized soil from Farm A (FA—light orange), unfertilized soil from Farm B (UB—dark salmon), and fertilized soil from Farm B (FB—light salmon). The bars represent the standard deviation. Asterisks indicate significant differences among samples (parametric data were analyzed using one-way ANOVA; nonparametric data were analyzed using the Kruskal–Wallis test). Letters denote significant differences among samples, as determined by post hoc tests (Tukey’s test or Dunn’s test).

The OTUs classified at the genus level accounted for 45% of the sequences, totaling 610 identified genera. The most abundant genera are represented in Figure 6. Significant differences (*p* < 0.05) in the relative abundances of the genera *Gaiella*, *Nocardioides*, *Nitrospira*, *Bradyrhizobium*, *Flavobacterium*, *Pedomicrobium*, *Sphingomonas*, and *Microlunatus* were detected among the samples. *Microlunatus* sp. was less abundant in untreated soil samples from Farm A. No significant differences were found in *Sphingomonas* composition across samples when analyzed using Tukey’s test.

**Figure 7:**
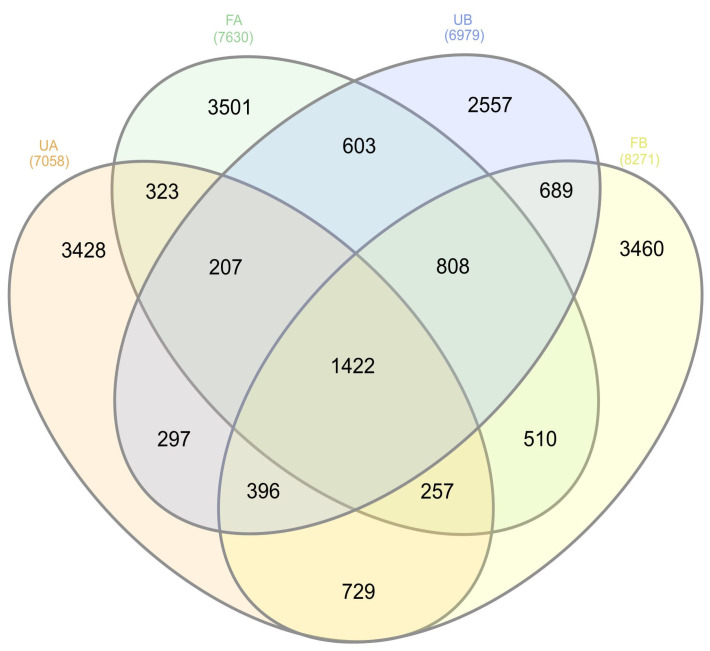
Venn diagram of OTUs shared among all the soil samples. The colors represent unfertilized soil from Farm A (UA—orange), fertilized soil from Farm A (FA—green), unfertilized soil from Farm B (UB—blue), and fertilized soil from Farm B (FB—yellow). The figure was generated using InteractiVenn [20].

In terms of bacterial community composition, OTUs were shared among samples, even between fertilized and unfertilized soils from different farms (Figure 7). Greater OTUs sharing was observed between poultry litter-fertilized and unfertilized soils at Farm B (689 OTUs) than at Farm A (323 OTUs).

## 3. Discussion

Antimicrobials from the fluoroquinolone, β-lactam, and sulfonamide classes, which are commonly used in animal production, are considered “critically important for human health” [14,16]. Antimicrobial resistance to these drugs has been increasingly reported in bacteria isolated from agricultural environments [12]. Given these concerns, our study aimed to evaluate the presence and distribution of ARB in poultry litter-fertilized and unfertilized soils.

In this study, although no statistically significant differences were observed among the samples, the CFU/g count of ciprofloxacin-resistant bacteria was slightly higher in soil fertilized with poultry litter from Farm A. Higher CFU/g counts of antimicrobial-resistant bacteria are expected in soils fertilized with fresh poultry litter, as this organic amendment can alter the soil microbiome, increasing both the abundance and diversity of ARGs and thereby shaping the soil resistome [21]. Additionally, the high concentrations of antimicrobials commonly present in animal-derived organic waste may contribute to the selection of resistant bacterial populations in the soil [11,22]. However, at Farm B, the CFU/g counts of ciprofloxacin-resistant bacteria were slightly higher in the unfertilized soil. Similarly, a slight increase in sulfamethoxazole-resistant CFU/g was observed in soils that had not been fertilized with poultry litter. However, these increases did not result in statistically significant differences. The comparable CFU/g counts in unfertilized soil may be associated with the influence of surrounding agricultural activities, in addition to the diversity of the soil resistome and the presence of bacteria with intrinsic resistance to these antimicrobials. Such resistance mechanisms may include alterations in membrane permeability, efflux pumps, inherently insensitive target enzymes, regulatory modifications of target enzymes, as well as mutations or recombination events affecting these enzymes [23,24].

Consequently, the ARGs investigated in this study were detected in bacterial strains isolated from both poultry litter-fertilized and unfertilized soils. Among the identified genes, *bla*_TEM_ and *bla*_SHV_ were the most frequently detected. The *bla*_TEM_ gene has already been recognized as widely disseminated in the environment. Owing to its broad geographic distribution, TEM-116, one of the main enzymes of the TEM family, is now considered a naturally occurring enzyme [25]. The *bla*_SHV_ gene has also been detected in various agricultural substrates, including manure, soil, and surface water [12,26]. In contrast, the *bla*_GES_ gene, which was identified in two *Bacillus* sp. strains isolated from unfertilized soil in Farm B, is less commonly found in environmental samples and is more frequently detected in Gram-negative bacilli, such as *Pseudomonas aeruginosa* and species of the Enterobacteriaceae family isolated from clinical settings [26,27]. However, previous studies have reported the presence of this gene in agricultural substrates where poultry litter was used. Lopes et al. [19] detected *bla*_GES_ in irrigation water from ponds around poultry farms. In another study, Lopes et al. [12] reported a rare occurrence of this gene in *Ochrobactrum* sp., *Stenotrophomonas* sp., and *Brevundimonas* sp. isolated from poultry litter and poultry litter-fertilized soil samples. These findings suggest the potential dissemination of this gene in agricultural environments associated with manure application.

β-Lactamase-encoding genes are often located on plasmids that also carry *qnr* genes [28]. In this study, the *qnrB* gene was detected alongside *bla*_TEM_ in *Citrobacter* sp. strains isolated from unfertilized soil at Farm B. The *qnrA* gene was identified in *Citrobacter* sp. strains from fertilized soil at Farm B, as well as in a *Bosea* sp. strain from unfertilized soil at Farm A, where it was detected along with *bla*_SHV_. The detection of these genes in *Citrobacter* sp. is not unexpected, as plasmid-mediated quinolone resistance (PMQR) genes are frequently associated with Enterobacteriaceae, particularly in pathogenic strains isolated from clinical environments. Fluoroquinolone and β-lactam resistance in Enterobacteriaceae is considered a widespread and urgent public health concern [29,30]. However, reports on the presence of *qnr* genes in *Bosea* sp. are scarce. Although *Bosea* sp. resistant to enrofloxacin has been reported in river biofilms [31] and ciprofloxacin-resistant strains have been identified in activated sludge [32], this genus, which belongs to the Alphaproteobacteria class, is common in soils but remains poorly studied [33].

The *intI1* and *intI2* genes were detected in *Bacillus* sp. (*intI1*) and *Pseudomonas* sp. (*intI1* and *intI2*). The presence of these genes suggests the occurrence of integrons, which are mobile genetic elements capable of acquiring, recombining, and expressing gene cassettes, including ARGs. Owing to these characteristics, integrons are strongly associated with the horizontal gene transfer (HGT) of resistance determinants, particularly among Gram-negative pathogens [34,35]. The *intI1* gene is also considered an environmental indicator of anthropogenic pollution and is widely distributed among commensal and pathogenic bacteria from both humans and animals [36]. In addition, the *sul1* gene is frequently found in conserved regions of class 1 integrons [37].

Interestingly, the *sul1* and *sul2* genes were not detected in this study. However, previous studies have reported the presence of these genes in strains isolated from different substrates within the same areas, including irrigation water, poultry litter-fertilized soil, the rhizosphere, sediment, and poultry litter itself [12,19]. Notably, none of the analyzed ARGs or class 1 and 2 integrases were detected in the total community DNA extracted from fertilized or unfertilized soils. These findings suggest that the target genes might be present at levels below the detection limit of the PCR technique, making their amplification challenging. Additionally, methodological limitations should be considered, such as the inherent sensitivity of PCR-based approaches. Alternatively, other genetic determinants may be involved in resistance to the antimicrobials studied, highlighting the complexity of resistance mechanisms in agricultural soils.

In terms of the structure and composition of the bacterial community in different soil types, alpha diversity was found to be similar across all the samples from both study areas. However, when only the soil type (poultry litter-fertilized vs. unfertilized soil) was considered, a significant increase in richness and diversity was observed in poultry litter-fertilized soil. This finding aligns with those of previous studies that evaluated the effects of poultry litter application on the soil microbiome [38,39].

While alpha diversity was relatively stable, beta diversity analyses revealed key differences in microbiome structure between fertilized and unfertilized soils. Regarding beta diversity, the distance between fertilized and unfertilized soil samples in Area A, as observed in the NMDS ordination, indicates differences in microbiome structure between the two soil types. Although the one-way PERMANOVA revealed a significant difference among all samples, in Farm B, this distinction is less evident, suggesting that the microbiome structure between fertilized and unfertilized samples in this area is more similar than that in Area A. Considering that both farms are located in the same region, share the same soil classification, and follow a similar agricultural pattern, this difference may be related to the spatial proximity between fertilized and unfertilized soils. In Farm B, the unfertilized area was only 10 m from the fertilized area, whereas in Farm A, the unfertilized soil (UA) was collected approximately 200 m away from the fertilized area. Previous studies have reported the potential for soil particle dispersion and the transport of poultry litter residues through erosive processes [12,19]. Since both farms are situated in valley areas, soil erosion and irrigation could increase the risk of leaching and dissemination of fertilized soil material into the environment. Therefore, the greater proximity between fertilized and unfertilized soils in Farm B may have facilitated a higher dispersion flux between areas, contributing to the greater similarity in microbiome structure observed in this locality.

Examining the microbial community composition in the soils, a significant increase in Bacillota abundance was observed in the fertilized soil from Farm A, which was likely due to modifications introduced by poultry litter fertilization. Bacillota is among the most abundant phyla in the avian gastrointestinal tract. Xiao et al. [40] reported that 60% of the DNA sequences obtained from the duodenum, jejunum, ileum, and colon of broiler chickens belong to this phylum. In agreement with previous studies, Bacillota, along with Proteobacteria and Bacteroidetes, which were also among the most abundant phyla in this study, constitute approximately 90% of the chicken microbiota [41,42]. Consequently, these phyla dominate poultry manure microbiota [12,39,43], and their increased abundance has been observed in poultry litter-fertilized soils [12,38].

Additionally, Gemmatimonadetes abundance was significantly higher in fertilized soil from Farm B, further suggesting compositional changes due to soil fertilization. Liu et al. [44] reported a greater abundance of this phylum in soils fertilized with chicken manure than in unfertilized soils or in soils treated with swine and sheep manure. Moreover, they reported a decrease in Gemmatimonadetes abundance in soils treated with cattle manure and chemical fertilizers.

In terms of the relative abundance of bacterial genera, *Microlunatus* sp. was significantly less abundant in unfertilized soil samples from Farm A. Sun et al. [45] reported an increase in the abundance of this genus in soils treated with ciprofloxacin, similar to those found in manure-amended soils, suggesting that the bacterial community composition may be influenced by the application of manure containing antimicrobial residues.

Finally, more pronounced differences in bacterial community composition between fertilized and unfertilized soils were observed at Farm A. The closer proximity between the fertilized and unfertilized areas at Farm B may have facilitated the transfer of poultry litter between soils, as well as the dispersal of the bacterial community. In addition, Farm B presented a greater degree of OTUs sharing between poultry litter-fertilized and unfertilized soils.

Additionally, clinically relevant bacterial strains carrying ARGs were identified in both farms and soil types. Several of these strains are listed as priority pathogens by the World Health Organization (WHO) for guiding research, development, and strategies to prevent and control AMR [16]. It is essential to consider the natural soil resistome, which encompasses the collection of ARGs present in both pathogenic and nonpathogenic bacteria, including free-living environmental bacteria and commensals associated with other organisms [46,47,48].

In addition to the immediate presence of ARGs in soil microbiomes, environmental factors further shape their dissemination pathways, posing challenges for their containment. In agricultural environments, this resistome can be altered by the direct introduction of contaminants into the soil, such as the application of manure containing antimicrobial residues, or by the spread of resistant bacteria and genes through biotic and abiotic factors. Once in the soil, resistant bacteria and ARGs can disseminate through leaching, surface runoff, and the migration of various soil- and water-associated organisms that may act as vectors [12]. Additionally, wind can contribute to the dispersion of airborne bacteria. Clinically relevant *Staphylococcus* sp. strains resistant to multiple antimicrobials, including sulfamethoxazole, have been detected in air samples collected outside poultry farms [49]. These factors pose challenges in selecting negative controls for in situ studies, such as the present study, but provide valuable insights into the mechanisms underlying the spread of AMR.

These findings underscore the complexity of AMR dissemination in agricultural environments. Beyond the introduction of ARGs and ARB through manure application, their persistence and mobility in soil can influence plant-associated microbiomes. ARGs present in poultry litter can be transferred to plants via aerosol deposition onto plant surfaces (phyllosphere) and through uptake into plant tissues, such as roots [50,51]. Zhang et al. [51] reported the exchange of ARGs between poultry litter-amended soil, the rhizosphere, the root endosphere, and the leaf endosphere of lettuce (*Lactuca sativa*), demonstrating that ARGs from contaminated soil can be internalized by plants, increasing the risk of direct human exposure through the consumption of raw vegetables. Given the widespread use of organic fertilizers, developing effective management strategies is critical for mitigating the spread of AMR in agricultural settings. Further studies should focus on the persistence, mobility, and uptake dynamics of ARGs across different soil types and cropping systems, with direct implications for food safety and public health.

## 4. Materials and Methods

### 4.1. Study Area and Sampling

The study was conducted with samples collected from the municipality of São José do Vale do Rio Preto (SJVRP), which is located in the mountainous region of Rio de Janeiro State, Brazil (615 m.a.s.l. mean elevation) [5,11]. Rosi et al. [52] followed the Köppen–Geiger climate classification and described the region as having a Tropical Highland climate, characterized by dry winters and wet summers.

The study area was previously described by Lopes et al. [19], who highlighted the municipality as one of the largest producers of poultry and agricultural products in Rio de Janeiro State. In poultry farming, different antimicrobials are employed, including β-lactams (e.g., ceftiofur and amoxicillin) and fluoroquinolones (e.g., enrofloxacin). Fresh poultry litter is commonly used as a fertilizer for agricultural soils [12].

Soil sampling was conducted on two horticultural farms located in São José do Vale do Rio Preto (SJVRP), designated A (22°06′54.7” S; 42°57′03.5′′ W) and B (22°08′57.7” S; 42°52′54.2′′ W). The soil of the studied farms was classified by Parente et al. [21] as *Latossolo Vermelho* in the Brazilian soil classification system [53], corresponding to Typic Hapludox in the US system [54]. Both farms use fresh poultry litter for soil fertilization. Additionally, as observed throughout the SJVRP region, these areas have sloping terrain and are situated in agricultural valleys, which may facilitate the mobilization of poultry litter from fertilized soil, as described by Lopes et al. [19].

Samples from both poultry litter-fertilized (F) and unfertilized (U) soils were collected in triplicate, using a previously sterilized trowel, from the surface layer (0–10 cm depth), totaling six samples per farm. On both farms, the unfertilized soil was covered with grasses (Poaceae) (Figure S2A). The fertilized soil was collected from areas that had undergone recent fertilization and cultivated with *Sechium edule* (chayote) (Figure S2B). For chayote cultivation, approximately 9 kg of poultry litter is applied at planting for each plant, with reapplication occurring every two to three months. At Farm A, unfertilized soil (UA) samples were collected approximately 200 m away from the area fertilized with poultry litter. The fertilized and unfertilized areas were separated by a pond covering approximately 665.25 m^2^, with a perimeter of 101.85 m. At Farm B, unfertilized soil (UB) samples were collected approximately 10 m from the fertilized area. In both farms, the distance between replicates within each area was approximately 3 m.

### 4.2. Isolation of Bacterial Strains Resistant to Different Antimicrobial Agents

To isolate bacterial strains resistant to different antimicrobials, 10 g of composite samples from poultry litter-fertilized and unfertilized soils (a total of 4 samples) were homogenized in 100 mL of 0.85% saline solution and shaken for 1 h at 100 rpm and 28 °C. Serial dilutions ranging from 10^−1^–10^−3^ were prepared for each sample. Each dilution was plated on CHROMagar™ medium (BD Diagnostics) supplemented with 60 µg/mL sulfamethoxazole (Sigma^®^, Buchs, Switzerland) or 50 µg/mL ciprofloxacin (Sigma^®^, Saint Louis, MO, USA) [19,28] and incubated at 37 °C for 24 h. After incubation, the number of colony-forming units per gram (CFU/g) was determined. Morphologically distinct colonies were isolated and subcultured under the same conditions for subsequent identification.

### 4.3. Identification of Strains Isolated from Poultry Litter-Fertilized and Unfertilized Soils

The bacterial strains isolated from the soil samples were identified using matrix-assisted laser desorption/ionization time-of-flight mass spectrometry (MALDI-TOF/MS) with the Microflex 62 LT system (Bruker^®^ Daltonik GmbH, Bremen, Germany) following the manufacturer’s protocol.

To confirm the MALDI-TOF identification and identify strains with score values below 1.700, i.e., those that could not be reliably identified by this technique, molecular identification was performed. For this purpose, genomic DNA was extracted from the isolated strains using the Fungal/Bacterial Miniprep Kit (Zymo Research, Tustin, CA, USA). The *rrs* gene (encoding 16 rRNA) of each strain was amplified by polymerase chain reaction (PCR) using the pA/pH primers [55] (Table S3). The PCR products were then purified with the Wizard SV Gel and PCR Clean-Up System Kit (Promega Corporation, Madison, WI, USA). The concentration of the purified samples was measured with a Qubit 4 fluorometer (Thermo Fisher Scientific, Waltham, MA, USA). A minimum of 10 ng/µL of each purified sample was used for Sanger sequencing (SeqStudio™—Applied Biosystems, Thermo Fisher, Waltham, MA, USA) of a 16S rRNA fragment employing the pA primer.

For taxonomic classification, the 16S rRNA sequences of each strain were compared against the GenBank database using the BLASTN tool from the National Center for Biotechnology Information (NCBI), applying thresholds of 99% coverage and 98% identity. The coverage and identity values, along with the accession numbers of the NCBI database sequences aligned to the dataset, are detailed in the Supplementary Materials (Table S2).

### 4.4. Extraction of Total DNA from Soil Samples

For total DNA extraction, 500 mg of each replicate from triplicate samples of poultry litter-fertilized (F) and unfertilized (U) soils from the two horticultural areas were weighed, resulting in a total of 12 samples. DNA extraction was performed using the direct lysis method with the Fast DNA™ SPIN Kit for Soil (MP Biomedicals, Santa Ana, CA, USA) following the manufacturer’s protocol. The quality and concentration of the DNA samples were assessed with a NanoDrop ND-1000 spectrophotometer (Thermo Fisher Scientific, Waltham, MA, USA) and a Qubit 4 fluorometer (Thermo Fisher Scientific, Waltham, MA, USA).

### 4.5. Detection of Antimicrobial Resistance Genes

As described by Lopes et al. [19], the presence of ARGs in the total DNA from soil samples and in the DNA of strains isolated from the same soil samples was evaluated using simplex and multiplex PCR reactions. Primers were used to target the *intI1* and *intI2* genes [56], which encode class 1 and class 2 integrases, respectively; the *bla*_GES_, *bla*_SHV_, and *bla*_TEM_ genes [57,58], which encode resistance to β-lactams; the *sul1* and *sul2* genes [59], which encode resistance to sulfonamides; and the *qnrA*, *qnrB* and *qnrS* genes [60], which confer resistance to fluoroquinolones. These genes were amplified using DNA extracted from soil samples and isolated strains. The sequences of the primers and the amplification parameters used are detailed in the Supplementary Materials (Table S3).

### 4.6. Analysis of the Total Bacterial Community in the Soil Samples Through Amplicon Sequencing of the Gene Encoding 16S rRNA

The structure and composition of the total bacterial community present in the poultry litter-fertilized (F) and unfertilized (U) soil samples, which were collected in triplicate from farms A and B, were determined through sequencing of the V3-V4 variable regions of the *rrs* gene encoding 16S rRNA. Paired-end sequencing was performed using the NovaSeq 6000 system (Illumina, San Diego, CA, USA). Library preparation was performed with the primers 341F (CCTAYGGGRBGCASCAG) and 806R (GGACTACNNGGGTATCTAAT) [61], which generate amplicons of approximately 450 bp. The sequences obtained from sequencing were analyzed with Mothur software v.1.48.0 [62], following the guidelines of the Illumina MiSeq SOP and as described by Lopes et al. [19]. Statistical analyses of the sequencing data, including alpha (α) and beta (β) diversity indices and relative taxonomic abundance, were conducted as described in Section 4.7.

The raw sequence data have been deposited in the NCBI Sequence Read Archive (SRA) and are available under the Bioproject accession number PRJNA1207078.

### 4.7. Statistical Analyses

Statistical analyses were performed using PAST v4.03 and Jamovi v2.3.28 [63,64]. All datasets were tested for normality using the Shapiro–Wilk test and for homoscedasticity using Levene’s test.

Alpha diversity (α-diversity), including species richness and diversity indices, was analyzed considering two factors: “area” (Farms A and B) and “treatment” (untreated soil and soil treated with poultry litter). When only one factor was analyzed, Student’s *t* test was applied to parametric, while the Mann–Whitney U test was used for nonparametric data. When both factors (“area” and “treatment”) were considered together, they were treated as independent variables, and parametric data were analyzed using one-way analysis of variance (ANOVA), whereas nonparametric data were analyzed using the Kruskal–Wallis test. Post hoc tests, such as Tukey’s test (parametric) and Dunn’s test (nonparametric), were subsequently applied as appropriate.

Beta diversity (β-diversity) was assessed using nonmetric multidimensional scaling (NMDS) based on Bray–Curtis dissimilarity index. Statistical differences in β-diversity among sample groups were tested using one-way PERMANOVA, based on the OTUs matrix.

Taxonomic composition and relative abundance analyses were conducted at the phylum and genus levels. Differences in relative abundance between groups were tested using one-way ANOVA for parametric data and the Kruskal–Wallis test for nonparametric data. When significant differences were detected, Tukey’s test (parametric) or Dunn’s test (nonparametric) was applied for multiple comparisons.

## 5. Conclusions

This study provides evidence that the use of poultry litter as fertilizer can influence the structure and composition of the soil microbiome, potentially increasing alpha diversity and enriching specific taxa such as Gemmatimonadetes and Bacillota. These findings also indicate the presence of antimicrobial-resistant bacteria (ARB) and resistance genes (ARGs) in both fertilized and unfertilized soils, highlighting the environmental persistence and potential spread of resistance elements. However, further studies with well-defined control areas are needed to better establish the causal relationship between poultry litter application and antimicrobial resistance dissemination. These findings reinforce the importance of improved management strategies to mitigate the spread of resistance in agricultural ecosystems. Monitoring AMR and understanding environmental resistomes through both culture-dependent and culture-independent methods are essential for developing effective mitigation measures. Strict regulatory controls on antimicrobial use in livestock, along with the investigation of efficient treatment strategies for poultry litter and other organic residues, represent key approaches to minimize AMR dissemination in agricultural settings.

## Figures and Tables

**Table 1:** Presence (+) and absence (−) of PCR amplification of genes conferring antimicrobial resistance in the DNA of strains isolated from poultry litter-fertilized (FA and FB) and unfertilized (UA and UB) soils from farms A and B.

Strain	Identification	Origen	*sul1*	*sul2*	*int1*	*int2*	*qnrA*	*qnrB*	*qnrS*	*bla*_TEM_	*bla*_GES_	*bla*_SHV_
**SULFAMETHOXAZOLE**												
SSFA5.2	*Bacillus* sp.	FA	−	−	−	−	−	−	−	*+*	−	−
SSUA3.1	*Klebsiella* sp.	UA	−	−	−	−	−	−	−	−	−	+
SSUA3.2	*Klebsiella variicola*	UA	−	−	−	−	−	−	−	+	−	+
SSUA4.1	*Pseudomonas* sp.	UA	−	−	+	+	−	−	−	−	−	−
SSUA12	*Klebsiella* sp.	UA	−	−	−	−	−	−	−	−	−	+
SSFB3.1	*Bacillus* sp.	FB	−	−	−	−	−	−	−	+	−	−
SSFB5.2	*Bacillus* sp.	FB	−	−	+	−	−	−	−	−	−	−
SSFB9.1	*Citrobacter* sp.	FB	−	−	−	−	+	−	−	+	−	−
SSFB12	*Citrobacter* sp.	FB	−	−	−	−	+	−	−	−	−	−
SSUB4.1	*Bacillus* sp.	UB	−	−	−	−	−	−	−	+	−	−
SSUB4.2	*Bacillus* sp.	UB	−	−	−	−	−	−	−	+	+	−
SSUB5.1	*Lynsinibacillus* sp.	UB	−	−	−	−	−	−	−	+	−	−
SSUB6.1	*Bacillus* sp.	UB	−	−	−	−	−	−	−	+	+	−
SSUB11	*Citrobacter* sp.	UB	−	−	−	−	−	+	−	+	−	−
SSUB13	Proteobacteria	UB	−	−	−	−	−	−	−	+	−	−
**CIPROFLOXACIN**												
CSUA1.2	*Bosea* sp.	UA	−	−	−	−	−	−	−	+	−	+
CSUA4.1	*Bosea* sp.	UA	−	−	−	−	+	−	−	−	−	+
CSUB5	*Stenotrophomonas* sp.	UB	−	−	−	−	+	−	−	−	−	−
CSUB8	*Stenotrophomonas* sp.	UB	−	−	−	−	+	−	−	−	−	−
CSFB1.2	*Microbacterium* sp.	FB	−	−	−	−	−	−	−	+	−	−
CSFB5.1	*Bosea* sp.	FB	−	−	−	−	−	−	−	−	−	+

## Data Availability

The raw data presented within this study are available upon request.

## Supplementary Materials

The following supporting information can be downloaded at: https://www.mdpi.com/article/10.3390/antibiotics14040355/s1, Figure S1: Individual rarefaction curves for all collected samples. Different colors represent distinct sample types: unfertilized soil from Farm A (UA—gold), unfertilized soil from Farm B (UB—red), fertilized soil from Farm A (FA—light yellow), and fertilized soil from Farm B (FB—light pink); Figure S2: Sampling. (**A**) Unfertilized soil with poultry litter. (**B**) Soil cultivated with *Sechium edule* (chayote) and fertilized with poultry litter. (**C**) Poultry farming sheds in area A. Original photographs taken during this study; Table S1: MALDI-TOF identification of strains grown on CHROMagar supplemented with 60 µg/mL sulfamethoxazole or 50 µg/mL ciprofloxacin; Table S2: Molecular identification: 16S rRNA gene sequencing of isolated strains; Table S3: Primers used for the amplification of gene encoding 16S rRNA, antimicrobial resistance genes and genes encoding integrases.
