# Supplementary material for: Microbiome and Resistome in Poultry Litter-Fertilized and Unfertilized Agricultural Soils
Source: Antibiotics (Basel). 2025 Mar 31;14(4):355. doi: 10.3390/antibiotics14040355 (PMC12024301; doi:10.3390/antibiotics14040355)
Supplement: Supplementary file 1 [file antibiotics-14-00355-s001.zip › antibiotics-3497319-supplementary.pdf]

antibiotics

MDPI

# Supplementary Materials

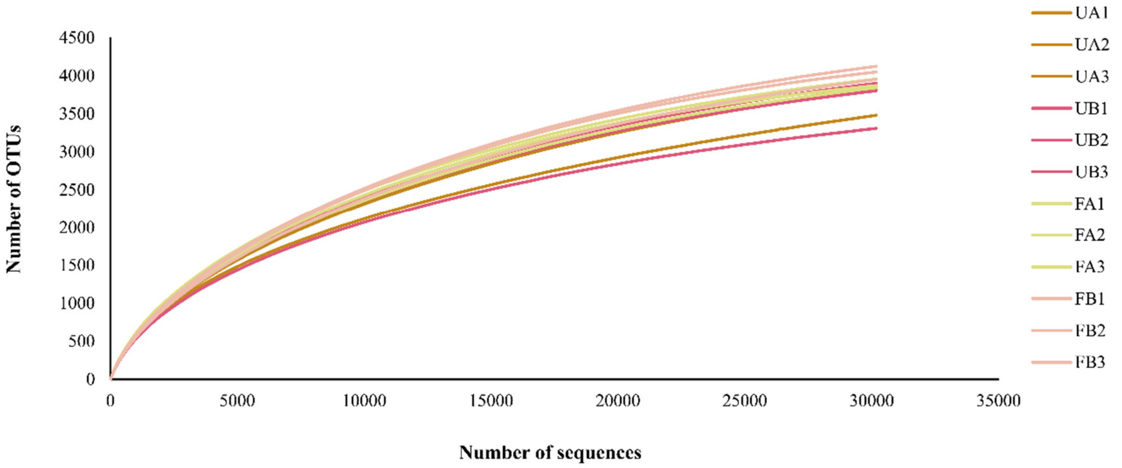


**Figure S1.** Individual rarefaction curves for all collected samples. Different colors represent distinct sample types: unfertilized soil from Farm A (UA – gold), unfertilized soil from Farm B (UB – red), fertilized soil from Farm A (FA – light yellow), and fertilized soil from Farm B (FB – light pink).

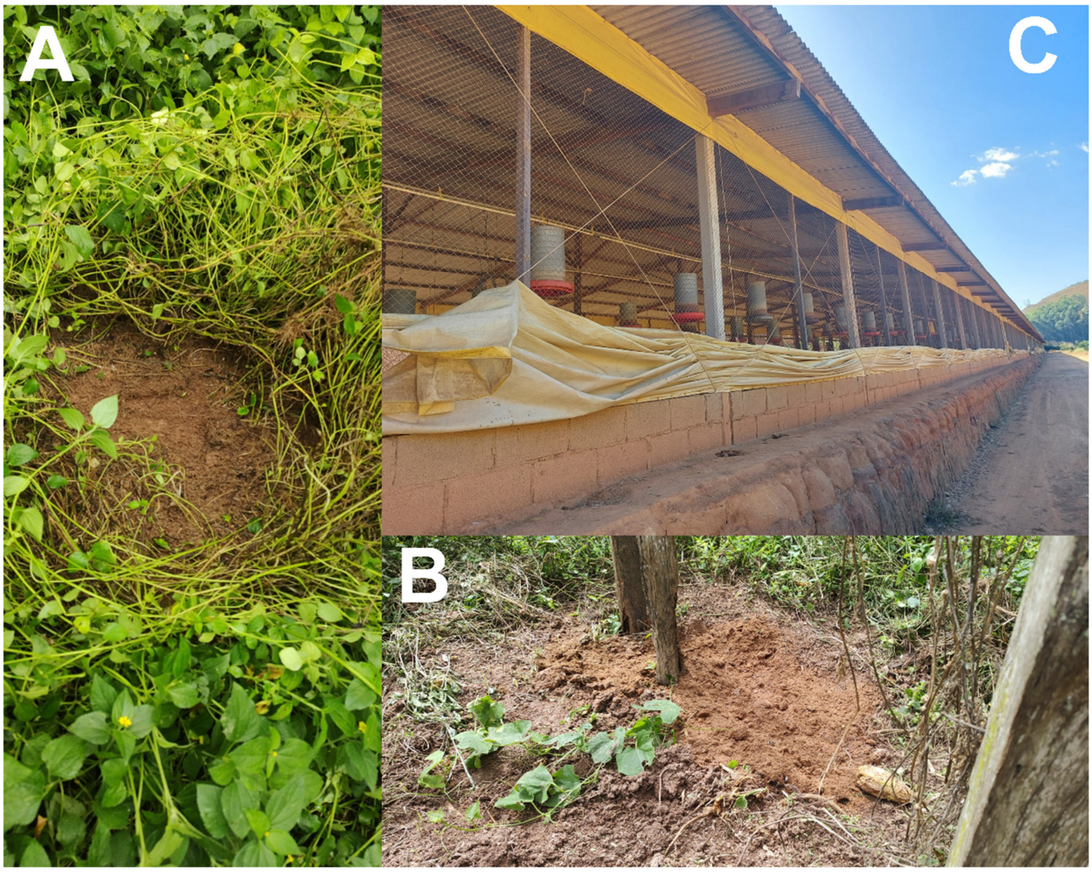


**Figure S2.** Sampling. **(A)** Unfertilized soil with poultry litter. **(B)** Soil cultivated with *Sechium edule* (chayote) and fertilized with poultry litter. **(C)** Poultry farming sheds in area A. Original photographs taken during this study.

**Table S1.** MALDI-TOF identification of strains grown on CHROMagar supplemented with 60 µg/mL sulfamethoxazole or 50 µg/mL ciprofloxacin.

| Identification | Sulfamethoxazole | | | | Ciprofloxacin | | | |
|---|---|---|---|---|---|---|---|---|
| | Farm A | | Farm B | | Farm A | | Farm B | |
| | SSUA | SSFA | SSUB | SSFB | CSUA | CSFA | CSUB | CSFB |
| *Mycoplasma arginni* | 0 | 2 | 0 | 0 | 0 | 0 | 0 | 0 |
| *Mycoplasma alkalescens* | 0 | 1 | 0 | 0 | 0 | 0 | 0 | 0 |
| *Salmonella* sp. | 0 | 1 | 0 | 0 | 0 | 0 | 0 | 0 |
| *Bacillus* sp. | 1 | 0 | 1 | 0 | 0 | 0 | 0 | 0 |
| *Bacillus cereus* | 0 | 1 | 1 | 7 | 0 | 0 | 0 | 0 |
| *Klebsiella* sp. | 1 | 0 | 0 | 0 | 0 | 0 | 0 | 0 |
| *Klebsiella variicola* | 2 | 0 | 0 | 0 | 0 | 0 | 0 | 0 |
| *Elizabethkingia miricola* | 1 | 0 | 0 | 0 | 0 | 0 | 0 | 0 |
| *Citrobacter* sp. | 0 | 0 | 1 | 1 | 0 | 0 | 0 | 0 |
| *Citrobacter braakii* | 0 | 0 | 0 | 1 | 0 | 0 | 0 | 0 |
| *Aeromonas hydrophila* | 0 | 0 | 1 | 1 | 0 | 0 | 0 | 0 |
| *Pectobacterium atrosepticum* | 0 | 0 | 0 | 1 | 0 | 0 | 0 | 0 |
| *Pectobacterium* | 0 | 0 | 1 | 0 | 0 | 0 | 0 | 0 |
| *Stenotrophomonas maltophilia* | 0 | 0 | 0 | 0 | 0 | 0 | 2 | 0 |
| *Microbacterium testaceum* | 0 | 0 | 0 | 0 | 0 | 0 | 0 | 1 |
| *Brevundimonas* sp. | 0 | 0 | 0 | 0 | 0 | 0 | 0 | 1 |
| *Enterobacter* sp. | 0 | 0 | 0 | 1 | 0 | 0 | 0 | 0 |
| Total number of identified strains | 5 | 6 | 5 | 12 | 0 | 0 | 2 | 2 |
| Total number of unclassified strains | 5 | 9 | 2 | 3 | 5 | 1 | 1 | 5 |

**Table S2.** Molecular identification: 16S rRNA gene sequencing of isolated strains.

| Strain | MALDI-TOF identification | 16S rRNA Gene Identification – Sequence with Highest Similarity | Aligned Fragment Size Compared to Database (bp) | identity (%) | Coverage (%) | Access Number | Taxonomic consensus |
|---|---|---|---|---|---|---|---|
| SSFA5.2 | Unclassified strain | *Bacillus wiedmannii* | 316 | 99.36 | 99 | NR_152692.1 | *Bacillus* sp. |
| SSUA3.1 | *Klebsiella variicola* | *Klebsiella pneumonia* | 336 | 98.27 | 100 | NR_037084.1 | *Klebsiella* sp. |
| SSUA3.2 | *Klebsiella variicola* | *Klebsiella variicola* | 232 | 100.00 | 100 | NR_025635.1 | *Klebsiella variicola* |
| SSUA4.1 | Unclassified strain | *Pseudomonas mosselli* | 556 | 99.64 | 100 | NR_024924.0 | *Pseudomonas* sp. |
| SSUA12 | *Klebsiella* sp. | *Klebsiella quasivariicola* | 287 | 97.90 | 100 | NR_181901.1 | *Klebsiella* sp. |
| SSFB3.1 | Unclassified strain | *Bacillus wiedmannii* | 196 | 96.92 | 98 | NR_152692.1 | *Bacillus* sp. |
| SSFB5.2 | *Bacillus cereus* | *Bacillus wiedmannii* | 401 | 99.75 | 100 | NR_152692.1 | *Bacillus* sp. |
| SSFB9.1 | *Citrobacter* sp. | *Citrobacter cronae* | 222 | 98.65 | 100 | NR_170426.1 | *Citrobacter* sp. |
| SSFB12 | *Citrobacter braaki* | *Pseudescharischia vulneris* | 345 | 91.88 | 100 | NR_041927.1 | *Citrobacter* sp. |
| SSUB4.1 | Unclassified strain | *Bacillus pacificus* | 460 | 99.35 | 100 | NR_157733.1 | *Bacillus* sp. |
| SSUB4.2 | *Bacillus cereus* | *Bacillus pacificus* | 430 | 99.77 | 100 | NR_157733.1 | *Bacillus* sp. |
| SSUB5.1 | Unclassified strain | *Lynsinibacillus xylanilyticus* | 565 | 97.70 | 100 | NR_116698.1 | *Lynsinibacillus* sp. |
| SSUB6.1 | *Bacillus* sp. | *Bacillus mobilis* | 748 | 99.45 | 100 | NR_157731.1 | *Bacillus* sp. |
| SSUB11 | *Citrobacter* sp. | *Citrobacter tructae* | 427 | 98.12 | 100 | NR_180641.1 | *Citrobacter* sp. |
| SSUB13 | *Pectobacterium* sp. | *Paenochrobactrum gallinarii* | 390 | 95.38 | 100 | NR_116966.1 | Proteobacteria |
| CSUA1.2 | Unclassified strain | *Bosea thiooxidans* | 499 | 98.00 | 100 | NR_041994.1 | *Bosea* sp. |
| CSUA4.1 | Unclassified strain | *Bosea thiooxidans* | 473 | 98.52 | 100 | NR_041994.1 | *Bosea* sp. |
| CSUB5 | *Stenotrophomonas maltophilia* | *Stenotrophomonas geniculata* | 378 | 99.47 | 100 | NR_024708.1 | *Stenotrophomonas* sp. |
| CSUB8 | *Stenotrophomonas maltophilia* | *Stenotrophomonas geniculata* | 590 | 98.99 | 100 | NR_024708.1 | *Stenotrophomonas* sp. |
| CSFB1.2 | Unclassified strain | *Microbacterium testaceum* | 454 | 99.34 | 100 | NR_026163.1 | *Microbacterium* sp. |
| CSFB5.1 | Unclassified strain | *Bosea robiniae* | 383 | 87.99 | 100 | NR_108516.1 | *Bosea* sp. |

**Table S3.** Primers used for the amplification of gene encoding 16S rRNA, antimicrobial resistance genes and genes encoding integrases.

| Gene | Primers | Sequence (5′-3′) | Reference | Amplification conditions |
|---|---|---|---|---|
| *rrs* | pA | AGAGTTTGATCCTGGCTCAG | [55] | 95°C - 3 min; 35 X (95°C - 1 min 48°C - 30 s; 72°C - 2 min); 72°C - 6 min |
| | pH | AAGGAGGTGATCCAGCCGCA | | |
| *intI*1 | intM1-UF | ACGAGCGCAAGGTTTCGGT | [56] | 94ºC - 10 min; 30 X (94º - 30 sec; 53º - 30 sec; 72º - 2 min); 72º - 7min |
| | intM1-DR | GAAAGGTCTGGTCATACATG | | |
| *intI*2 | intM2-UF | GTGCAACGCATTTTGCAGG | | |
| | intM2-DR | CAACGGAGTCATGCAGATG | | |
| *bla*SHV | mSHV-F | CTTGACCGCTGGGAAACGG | [57,58] | 95ºC - 10 min; 30 X (95º - 30 sec; 55º - 30 sec; 72º - 45 sec); 72º - 10 min |
| | mSHV-R | AGCACGGAGCGGATCAACGG | | |
| *bla*TEM | mTEM-F | CCCTTATTCCCTTTYTTGCGG | | |
| | mTEM-R | AACCAGCCAGCCWGAAGG | | |
| *bla*GES | mGES-F | AGCAGCTCAGATCGGTGTTG | | |
| | mGES-R | CCGTGCTCAGGATGAGTTG | | |
| *sul1* | Sul1-F | GAATAAATCGCTCATCATTTTCGG | [59] | 95ºC - 10 min; 30 X (95º - 1 min; 52º - 45 sec; 72º - 1 min); 72º - 1min |
| | Sul1-R | CGAATTCTTGCGGTTTCTTTCAGC | | |
| *sul2* | Sul2-F | ATGGTGACGGTGTTCGGCATTCTGA | [59] | 95ºC - 10 min; 30 X (95º - 1 min; 55º - 45 sec; 72º - 1 min); 72º - 1min |
| | Sul2-R | CTAGGCATGATCTAACCCTCGGTCT | | |
| *qnrA* | QnrAm-F | AGAGGATTTCTCACGCCAGG | [60] | 95ºC - 10 min; 25 X (95º - 45 sec; 58º - 45 sec; 72º - 15 sec); 72º - 3 min |
| | QnrAm-R | TGCCAGGCACAGATCTTGAC | | |
| *qnrS* | QnrSm-F | GCAAGTTCATTGAACAGGGT | | |
| | QnrSm-R | TCTAAACCGTCGAGTTCGGCG | | |
| *qnrB* | QnrBm-F | GGMATHGAAATTCGCCACTG* | | |
| | QnrBm-R | TTTGCYGYYCGCCAGTCGAA* | | |

*M = A or C; H = A or C or T; Y = C or T.

## References

55. Massol-Deya A.A.; Odelson D.A.; Hickey R.F. Bacterial Community Fingerprinting of Amplified 16S and 16–23S Ribosomal DNA Gene Sequences and Restriction Endonuclease Analysis (ARDRA). In *Mol Micro Eco Manual*; Akkermans, A.D.L., Van Elsas, J.D., De Bruijn, F.J., Eds.; Springer Netherlands: Dordrecht, 1995; pp. 289–296 ISBN 978-94-010-4156-0.

56. Xu, X.; Kong, F.; Cheng, X.; Yan, B.; Du, X.; Gai, J.; Ai, H.; Shi, L.; Iredell, J. Integron Gene Cassettes in *Acinetobacter* spp. Strains from South China. *Inter J Anti Ag* 2008, *32*, 441–445, doi:10.1016/j.ijantimicag.2008.05.014.

57. Campana, E.H.; Xavier, D.E.; Petrolini, F.V.-B.; Cordeiro-Moura, J.R.; Araujo, M.R.E. de; Gales, A.C. Carbapenem-Resistant and Cephalosporin-Susceptible: A Worrisome Phenotype among *Pseudomonas aeruginosa* Clinical Isolates in Brazil. *The Braz J Inf Dis* 2017, *21*, 57–62, doi:10.1016/j.bjid.2016.10.008.

58. Picão, R.C.; Poirel, L.; Gales, A.C.; Nordmann, P. Diversity of β-Lactamases Produced by Ceftazidime-Resistant *Pseudomonas aeruginosa* Isolates Causing Bloodstream Infections in Brazil. *Anti Ag Chemo* 2009, *53*, 3908–3913, doi:10.1128/AAC.00453-09.

59. Toleman, M.A.; Bennett, P.M.; Bennett, D.M.C.; Jones, R.N.; Walsh, T.R. Global Emergence of Trimethoprim/Sulfamethoxazole Resistance in *Stenotrophomonas maltophilia* Mediated by Acquisition of *sul* Genes. *Emerg Inf Dis* 2007, *13*, 559–565, doi:10.3201/eid1304.061378.

60. Cattoir, V.; Poirel, L.; Rotimi, V.; Soussy, C.-J.; Nordmann, P. Multiplex PCR for Detection of Plasmid-Mediated Quinolone Resistance *qnr* Genes in ESBL-Producing Enterobacterial Isolates. *J Anti Chemo* 2007, *60*, 394–397, doi:10.1093/jac/dkm204.
